# Development of artificial neural network models for paediatric critical illness in South Africa

**DOI:** 10.3389/fped.2022.1008840

**Published:** 2022-11-15

**Authors:** Michael A. Pienaar, Joseph B. Sempa, Nicolaas Luwes, Elizabeth C. George, Stephen C. Brown

**Affiliations:** ^1^ Paediatric Critical Care Unit, Department of Paediatrics and Child Health, University of the Free State, Bloemfontein, South Africa; ^2^ Department of Biostatistics, Faculty of Health Sciences, University of the Free State, Bloemfontein, South Africa; ^3^ Department of Electrical, Electronic and Computer Engineering, Faculty of Engineering, Built Environment and Information Technology, Central University of Technology, Bloemfontein, South Africa; ^4^ Medical Research Council Clinical Trials Unit, University College London, London, United Kingdom; ^5^ Paediatric Cardiology Unit, Department of Paediatrics and Child Health, University of the Free State, Bloemfontein, South Africa

**Keywords:** neural networks, machine learning, critical care, children, triage, severity of illness

## Abstract

**Objectives:**

Failures in identification, resuscitation and appropriate referral have been identified as significant contributors to avoidable severity of illness and mortality in South African children. In this study, artificial neural network models were developed to predict a composite outcome of death before discharge from hospital or admission to the PICU. These models were compared to logistic regression and XGBoost models developed on the same data in cross-validation.

**Design:**

Prospective, analytical cohort study.

**Setting:**

A single centre tertiary hospital in South Africa providing acute paediatric services.

**Patients:**

Children, under the age of 13 years presenting to the Paediatric Referral Area for acute consultations.

**Outcomes:**

Predictive models for a composite outcome of death before discharge from hospital or admission to the PICU.

**Interventions:**

None.

**Measurements and main results:**

765 patients were included in the data set with 116 instances (15.2%) of the study outcome. Models were developed on three sets of features. Two derived from sequential floating feature selection (one inclusive, one parsimonious) and one from the Akaike information criterion to yield 9 models. All developed models demonstrated good discrimination on cross-validation with mean ROC AUCs greater than 0.8 and mean PRC AUCs greater than 0.53. ANN1, developed on the inclusive feature-et demonstrated the best discrimination with a ROC AUC of 0.84 and a PRC AUC of 0.64 Model calibration was variable, with most models demonstrating weak calibration. Decision curve analysis demonstrated that all models were superior to baseline strategies, with ANN1 demonstrating the highest net benefit.

**Conclusions:**

All models demonstrated satisfactory performance, with the best performing model in cross-validation being an ANN model. Given the good performance of less complex models, however, these models should also be considered, given their advantage in ease of implementation in practice. An internal validation study is now being conducted to further assess performance with a view to external validation.

## Introduction

The reduction of avoidable childhood mortality and morbidity is a key healthcare priority worldwide. Steps to address failures in acute care and critical care processes are an important aspect of strategies for improving care for critically ill or injured children (1). One such failure is within triage and identification systems needed to detect children in need of life-saving care (2). In South Africa, Hodkinson et al. found that patients with critical illness follow complex pathways to appropriate care and that failures in identification, accurate assessment of the severity of illness, early resuscitation, and timely referral to higher levels of care were responsible for significant avoidable severity of illness and mortality (3). This serves as the rationale for conducting research in this area and developing clinically implementable tools for the identification of critically ill children that are appropriate to the South African setting.

Triage scores are directed at sorting patients into categories of urgency based on a combination of clinical characteristics (history and clinical discriminators) and physiological variables (4, 5). The purpose of these systems is to optimise patient waiting times and ensure patients receive appropriate and timeous treatment. Several triage systems have been implemented in South Africa, although, in some regions, no formal triage is applied (6). These include the South African Triage Scale (SATS), Early Triage Assessment and Treatment (ETAT) program and the Integrated Management of Childhood Illness (IMCI) program (5–9). These systems are implemented by a variety of healthcare workers, but nurse-based triage is frequently the arrangement in South Africa (5). Despite the presence of triage systems in South Africa, the findings of Hodkinson et al., illustrate that the identification of patients requiring referral to centralised services remains a key contributor to avoidable death and severity of illness (3).

The role of applied machine learning in medicine and, more specifically in paediatrics and child health has, as Lonsdale and Rajkomar point out, not been fully explored in the literature (10, 11). Machine learning offers a wide range of possible use-cases in the clinical setting, including diagnosis, prognosis, workflow and improving patient access to services (11).

The use of machine learning in triage and mortality prediction has been described in high-income countries. Goto et al. and Hwang and Lee have described the use of machine learning to predict paediatric intensive care unit (PICU) admission and hospitalisation in the Emergency Department setting and critical illness respectively (12, 13). Aczon et al. and Kim et al. have described models for the prediction of mortality in PICU. Notably, these models represent significant progress compared to existing models in their ability to make dynamic or continuous assessments of mortality risk over time (14, 15). Our unit has recently published the development of an artificial neural network(ANN) model for PICU mortality risk prediction with comparable performance to a range of logistic regression models (16). With this rationale, the development of an ANN model designed to identify children at risk of severe illness requiring urgent intervention or escalation of care to a PICU was undertaken. Several distinctions exist between this study and our prior work. Our PICU mortality risk prediction model was designed to evaluate quality of care against model predictions of mortality, whereas the models developed in this study are intended to improve identification of children with or at risk of critical illness at the time of presentation. The models developed in this study are intended to augment the ability of clinicians with limited experience and expertise in paediatric critical illness in resource constrained settings. As such, the variables selected for use in these models were identified using a dedicated domain knowledge elicitation process including a Delphi procedure and systematic literature search (17). The variables identified were selected on the grounds that they represented a compromise between comprehensiveness and ease of use and specifically excluded radiological and laboratory variables except point of care glucose testing. This contrasts with the variables included in PICU mortality prediction models, where more extensive and advanced data is typically available or acquired at the time of admission to PICU.

ANNs are computational structures that broadly simulate the learning process and organisation of biological neurons (18). The network architecture is made up of interconnected input, hidden and output layers. The strength or intensity of each connection is represented by a numerical value, the weight, and each neuron has a threshold term for activation. Outputs of neurons are determined by a mathematical function, the activation function that takes in the inputs to input neurons, weights of connections and threshold terms of each neuron (19). Learning is achieved when weights are adjusted through stochastic gradient descent (20).

This model was compared to a logistic regression (LR) model and XGBoost model developed on the same data. The primary objective was to develop an artificial neural network(ANN) model for the prediction of this outcome.

## Methods and study design

**Figure 1:**
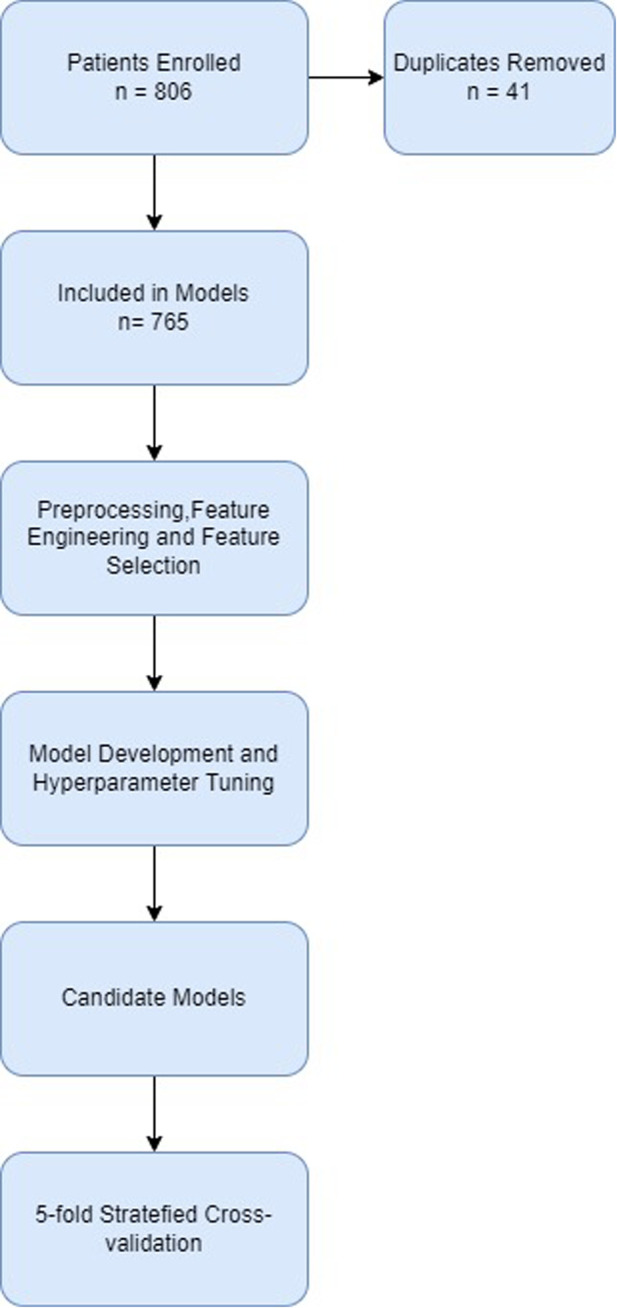
Consort diagram of development pipeline.

The development pipeline used in this study is summarized in Figure 1.

### Ethical clearance

Internal review board approval was obtained from the Health Sciences Research Ethics Committee (UFS-HSD2020/2204/2505-0003) and the Free State Department of Health (FS_202102_019). Informed consent was obtained in writing from the legal guardians of children participating in the study. Assent was obtained from children capable of doing so. All data were stored on a secure server and were fully anonymised before exporting as a CSV file for analysis.

### Study site

This study was conducted at Pelonomi Regional Hospital, a regional referral hospital in Mangaung in the Free State, South Africa. Data was collected in the Paediatric Referral Area. This area receives acute referrals for specialised paediatric consultations for the Southern Free State region and all patients hospitalised by the Paediatrics and Child Health service are first assessed in this area. Patients are referred from primary healthcare centres and district hospitals as well as accepting referrals from the Emergency Department. The Paediatrics and Child Health service is supported by a five-bedded PICU within Pelonomi Hospital that provides life-supporting therapies, including mechanical ventilation, cardiovascular support (excluding extracorporeal life support) and renal replacement therapy together with a full suite of invasive and non-invasive monitoring.

### Study population and sampling

Data collection was conducted from April 2021 to January 2022. The inclusion criteria were as follows:

1. Children under the age of 13 years
2. Unscheduled consultations
3. Duration of illness or injury less than 7 days (including acute exacerbations of underlying chronic illness)

Children presenting dead on arrival and admitted directly referred to the PICU were excluded as the model outcome was already present. Children presenting for scheduled clinic visits and elective procedures were also excluded from the study.

The minimum required size of the sample was estimated by considering the total number of records required as well as the number of events of the study outcome in relation to the number of features that can be included in the model without overfitting. The minimum sample size was determined by the rule of thumb of 500 and *n *= 100 + 50*i. n* refers to sample size and *i* to the number of features included in each model (21). The minimum number of events required per feature was set at 10 (22). For a model with ten features, a development sample of at least 600 samples was required with 100 events.

### Data collection

The process of determining which features (predictors or independent variables) were to be collected was determined by a domain knowledge elicitation process and described previously (17). The included features are presented in Supplementary Table S1.

Data collection was conducted prospectively at the bedside by the primary physician of each patient during initial assessment. Data collection made use of a REDCap® survey completed by the treating physician on their smartphone. Enrolled patients were monitored by the principal researcher until discharge from hospital or death and the outcome was entered into the research record.

### Data preprocessing

Data preprocessing was conducted before model development, training and testing. The first step was the removal of duplicate entries, followed by manual deletion of impossible values, e.g., systolic blood pressures lower than diastolic blood pressure. Anthropometric values, pulse rate, respiratory rate, systolic blood pressure and mean blood pressure (calculated from systolic and diastolic blood pressure) were normalised to available age-related norms as Z-scores and standard deviations and then converted to their absolute(positive) values (23–26). Further preprocessing was conducted within cross-validation to prevent information leakage from the test folds into training folds. Missing values were imputed by multiple imputation through chained equations (MICE) (27).

### Feature selection

Features were selected for inclusion in model training by sequential forward floating feature selection, using an ANN model as estimator (28) and Akaike Information Criterion (AIC) (29). Selected features are presented in Table 1. Features were considered by the researcher in terms of ambiguity and clinical relevance as well as the degree of correlation with the study outcome evaluated by correlation matrix visualisation. For this reason, the HIV status variable was removed from the set initially selected by sequential feature selection.

**Table 1:** Lists of features selected for inclusion in candidate models.

Inclusive Features	Parsimonious Features	AIC Features
Respiratory rate Z-score	Mean blood pressure Z-score	Respiratory Rate
Peripheral oxygen saturation	Capillary blood glucose	Peripheral oxygen saturation
Pulse rate	Respiratory distress	Capillary blood glucose
Mean blood pressure Z-score	AVPU scale	Level of consciousness
Capillary refill time	Inability to feed	Age
Capillary blood glucose		Inability to feed
Respiratory distress		
Weak pulses		
Level of consciousness		
Inability to feed		

### Feature engineering

Features were further optimised for model training. Some features represent clinical abnormalities above and below their normal values or in positive and negative ranges. Respiratory rate and mean blood pressure Z-scores were converted to their absolute (positive) values. Capillary blood glucose was separated into hyper- and hypoglycaemia features as continuous values greater than a threshold of abnormality. For level of consciousness and AVPU scale, because of the small number of records in some categories, the feature was reduced to a binary feature. Features were then scaled by standard scaling. Engineered features are presented in Table 2.

**Table 2:** Feature engineering.

Base Feature	Engineered Feature
Respiratory rate Z-score	Absolute respiratory rate Z-score
Mean blood pressure Z-score	Absolute Mean blood pressure Z-score
Capillary blood glucose	Hyperglycaemia:
Level of consciousness/AVPU	*<10 mmol/L: 0*
	*>= 10 mmol/L: Capillary blood glucose – 10*
	Hypoglycaemia:
	*>3 mmol/L: 0*
	*<= 3 mmol/L: 3 – capillary blood glucose*
	Alert:
	*Yes: 1*
	*No: 0*

### Class imbalance

In the presence of imbalanced data sets, models will tend to be biased towards the majority class (30–33). For the neural network models, class imbalance was addressed by employing focal loss as the loss parameter for training, whereby a focusing parameter(*γ*) reduces the loss from easy examples and a weighting factor(*α*) for each class (33). For the comparator models, sample weighting was employed to address class imbalance. These parameters were tuned during hyperparameter tuning.

### Model development

Model development was conducted within 5-fold stratified cross-validation. For each of the three feature-sets, an ANN model, LR model and XGBoost model were developed. This resulted in a total of 9 candidate models. The models were developed in Python 3 on the Jupyter Notebooks environment (34). The following libraries were used during the process: Pandas, Numpy, SciKit Learn, Mlxtend, Tensorflow, XGBoost, Statsmodels and Matplotlib (28, 35–41). An ANN, LR and XGBoost model was developed on each feature-set for comparison. Hyperparameters of the machine learning models were tuned by iteratively and by randomized grid search. Models were trained in five-fold stratified cross validation with a resultant 80:20 train-test split on each fold. Models were all trained with the same train-test splits.

### Model performance measurement

Model performance was assessed as mean performance in cross-validation. Discrimination was assessed in terms of the receiver operating characteristic (ROC) curve and the area under the curve (AUC) together with the precision-recall curve (PRC) with its AUC. Where the ROC curve relates recall or sensitivity to the false positive rate (1 − specificity), PRC relates precision (also called positive predictive value [True Positives/(True Positives + False Positives)] and recall [True Positives/(True Positives + False Negatives)] and provides a model-wide evaluation of performance. The AUC of the ROC and PRC allow comparisons between models. As opposed to the ROC AUC, where the baseline value for random classifiers is 0.5, the value for random classifiers in the case of the PRC AUC is not fixed, but rather corresponds to the proportion of positive class [Positive/(Positive + Negative)]. The PRC AUC of a perfect classifier is 1.0 (32). AUC 95% confidence intervals were calculated by a non-parametric bootstrap procedure for both PRC and ROC (42). Calibration was assessed by the means of the expected calibration error(ECE) for each model (43) together with the calibration hierarchy published by Van Calster et al. (44) – flexible calibration curves were plotted and inspected, and the slope and intercept of these curves were calculated.

The likely clinical utility of models was compared using decision curve analysis. Decision curve analysis was first described by Vickers and Elkin in 2006 (45). Decision curve analysis evaluates the “net benefit” of a predictive model or diagnostic test over a range of threshold probabilities and compares it to baseline strategies of responding in all cases or none. Net benefit is calculated as:

netbenefit=sensitivity×prevalence−(1−specificity)×(1−prevalence)×w

where *w* is the odds at the threshold probability (46).

## Results

### Data set

After duplicated records were removed, 765 records remained. 116 (15.2%) patients had the study outcome of death before discharge from hospital or admission to the PICU. The descriptive analysis of the collected data, together with the frequency of missing data is presented in Table 3. Continuous features are represented as means with standard deviations (SD) and categorical features are presented as frequencies and percentages.

**Table 3:** Descriptive analysis of data Set.

	Continuous Features			
	Unit	Mean	SD	Missing
Age	Months	30.61	39.65	
Respiratory Rate	Frequency	42.44	15.05	1
SPO_2_	%	96.19	7.00	
Pulse	Frequency	143.79	27.73	
Systolic Blood Pressure	mmHg	98.38	18.76	4
Diastolic Blood Pressure	mmHg	60.86	15.74	
Capillary Refill Time	Seconds	2.29	0.76	14
Weight	kg	10.46	8.16	13
Height	cm	78.07	27.03	39
Temperature	°C	36.94	0.93	3
Glucose	mmol/L	6.04	4.25	6
	**Categorical Features**			
	**Categories**	**Freq.**	**%**	**Missing**
Deep Breathing	No	544	71.58	5
	Yes	216	28.42	
Weak Pulse	No	713	93.20	
	Yes	52	6.80	
Level of Consciousness	Alert	666	87.06	
	Prostrate	89	11.63	
	Coma	10	1.31	
AVPU Scale	Alert	635	83.53	
	Verbal	56	7.97	
	Pain	61	7.32	
	Unresponsive	9	1.18	
Unable to Feed	No	568	74.25	
	Yes	197	25.75	
Respiratory Distress	No	519	67.84	
	Yes	246	32.16	
Jaundice	No	717	93.73	
	Yes	48	6.27	
Seizures	No	634	82.88	
	Yes	131	17.12	
Respiratory Support	Room Air	569	74.38	
	Nasal Cannula	185	24.18	
	Intubated	11	1.44	
HIV Infection	Unexposed	499	65.23	
	Exposed, uninfected	197	25.75	
	Infected	49	6.41	
	*Untreated*	10	1.31	
	*Treatment <3 months*	12	1.57	
	*Treatment >=3 months*	24	3.14	
	*Treatment Interrupted*	3	0.39	
	Unknown	20	2.61	
Outcome	Death	30	3.92	
	PICU admission	99	12.94	
	Combined outcome	116	15.16	

### Models

**Figure 2:**
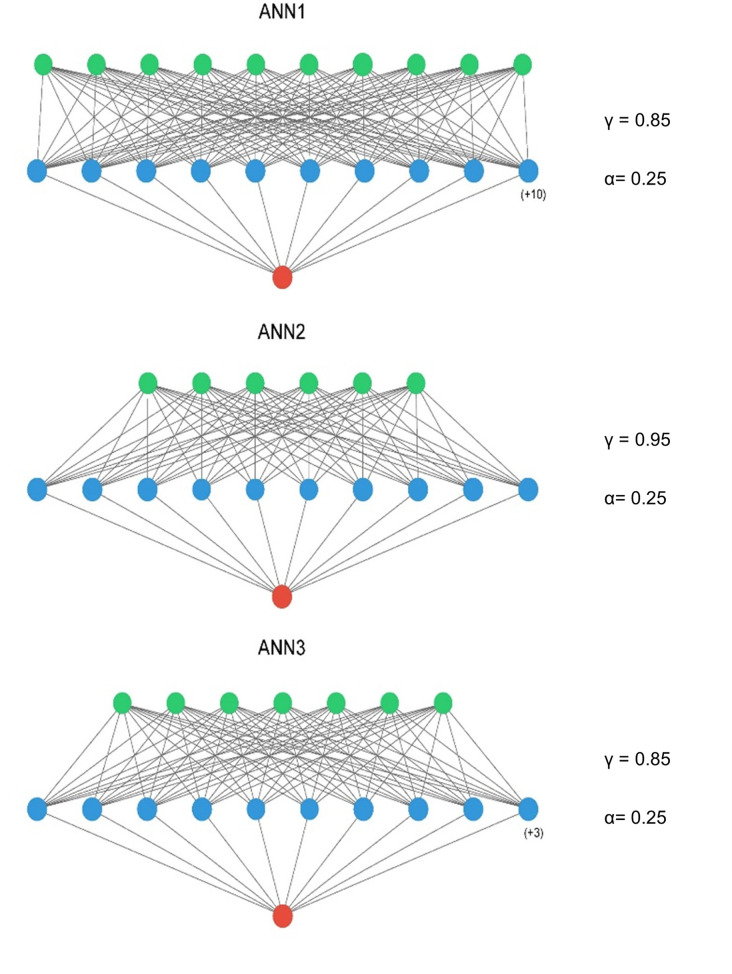
ANN model architectures – input layer = green, hidden layer = blue, output layer = red. A ReLu activation was used in the input and hidden layers and a sigmoid activation function in the output layer. ANN1 – Inclusive Model. ANN2 – Parsimonious Model, ANN3 – AIC Model. Figure compiled using ann_visualizer (51).

The optimised ANN architectures for each of the candidate neural networks are presented in Figure 2. All three models were simple feed-forward perceptrons with a single hidden layer. Batch normalisation was not used, in keeping with the observation that it negatively influences model calibration (47), and training epochs were set at 15 for all models. The tuned hyperparameters for the machine learning models are presented in Table 4.

**Table 4:** Tuned hyperparameters.

	ANN1	ANN2	ANN3
Hidden layers	1	1	1
Hidden layer activation function	ReLu	ReLu	ReLu
Hidden layer neurons	20	10	13
Output layer activation function	Sigmoid	Sigmoid	Sigmoid
Optimizer	Adam (learning rate = 0.001)	Adam (learning rate = 0.001)	Adam (learning rate = 0.001)
Loss Function	Focal Loss (*α* = 0.25, *γ* = 0.85)	Focal Loss (*α* = 0.25, *γ* = 0.95)	Focal Loss (*α* = 0.25, *γ* = 0.85)
Initializer	He Uniform	He Uniform	He Uniform
Batch Size	1	1	1
Epochs	15	15	15
	**XGB1**	**XGB2**	**XGB3**
Target Weight	1.4	1.4	1.4
Learning Rate	0.08	0.08	0.08
Subsample	0.9	0.8	0.8
Estimators	50	50	50
Alpha	0.2	0.2	0.2
Gamma	1.5	2.5	3.5

The logistic regression models for each feature-set are presented in Table 5.

**Table 5:** Logistic regression models.

Inclusive Model (LR1)				
** **		Coef.	*p*-value	CI
Intercept		−2.23	<0.001	−2.50, −1.94
Respiratory Rate Z-score		0.02	0.85	−1.20, 2.42
Peripheral Pulse Oximetry		−0.19	0.07	−0.39, −0.01
Pulse Rate		−0.13	0.26	−0.37, 0.10
Mean Blood Pressure Z-Score		0.21	0.08	−0.02, 0.43
Capillary Refill Time		0.03	0.84	−0.24, 0.30
Glucose	Hypoglycaemia	0.16	0.17	−0.07, 0.38
	Hyperglycaemia	0.50	<0.001	0.28,0.74
Alert		−0.40	<0.001	−0.62, −0.18
Weak Peripheral Pulse		0.12	0.30	−0.10, 0.36
Respiratory Distress		0.58	<0.001	0.33, 0.82
Unable to Feed		0.54	<0.001	0.30, 0.78
**Parsimonious Model (LR2)**				
** **		**Coef.**	***p*-value**	**CI**
Intercept		−2.22	<0.001	−2.50, −1.93
Mean Blood Pressure Z-Score		0.24	0.03	0.08,0.50
Glucose	Hypoglycaemia	0.23	0.02	0.03, 0.43
	Hyperglycaemia	0.51	0.51	0.28, 0.74
Alert		−0.48	<0.001	−0.69, −0.26
Respiratory Distress		0.60	<0.001	0.38, 0.83
Unable to Feed		0.49	<0.001	0.25, 0.74
**AIC Model (LR3)**				
** **		**Coef.**	***p*-value**	**CI**
Intercept		−2.16	<0.001	−2.43, −1.89
Peripheral Pulse Oximetry		−0.28	0.004	−0.48, −0.09
Glucose	Hypoglycaemia	0.21	0.10	0.00, 0.41
	Hyperglycaemia	0	<0.001	0.28, 0.71
Alert		−0.27	<0.001	−0.62, −0.07
Unable to Feed		0.59	<0.001	0.31, 0.80
Respiratory Rate		0.47	<0.001	0.24, 0.70
Age		0.13	0.33	0.00, 0.41

### Main results

**Figure 3:**
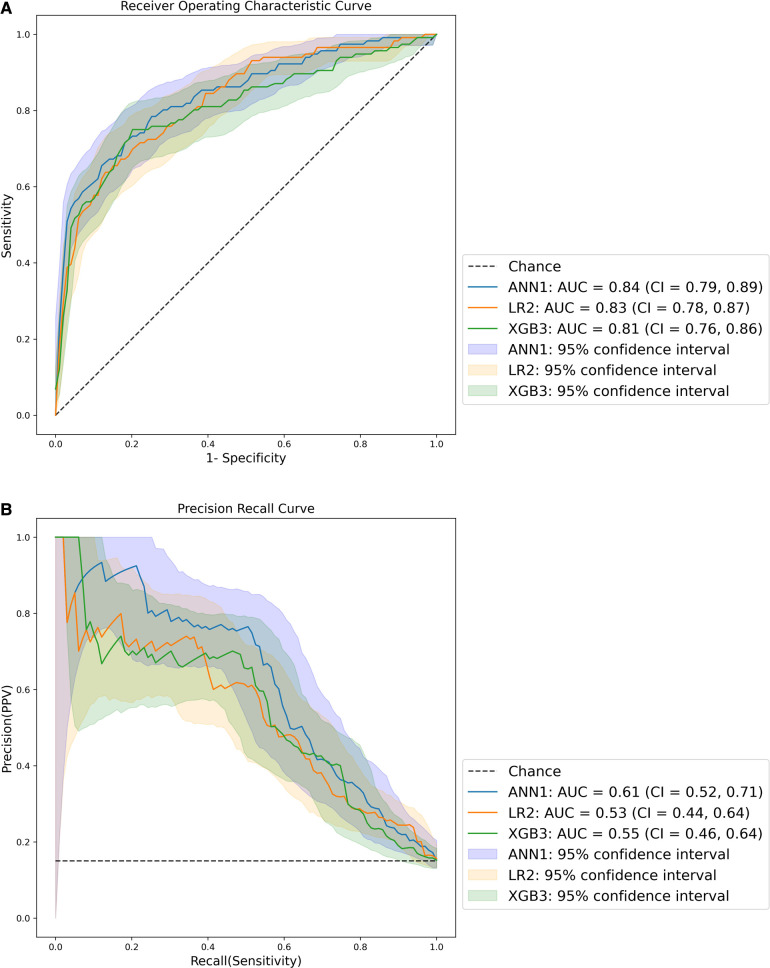
ROC (**A**) and PR (**B**) curves – AUC = area under the curve, CI = 95% confidence interval.

The ROC and PRC of the best performing models for each method are presented in Figure 3. The best performing model was ANN1, derived from the inclusive data set, with the highest ROC AUC and PRC AUC. All models had ROC AUCs of at least 0.8, indicating excellent discrimination (48). All models had PRC AUCs above 0.53, above the threshold for a random classifier (0.15). The curves for all models are presented in Supplementary Figure S1.

**Figure 4:**
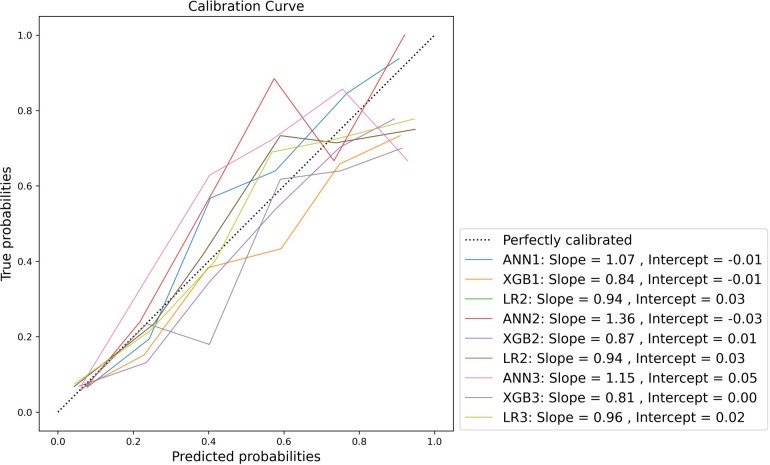
Normalised flexible calibration curve.

Calibration was variable across the models. Model-wide calibration was similar as assessed by ECE (see Table 6). Most models were weakly calibrated with calibration slopes close to 1 and intercepts close to 0. Inspection of the flexible calibration curves, however, suggests that models were generally not close to the perfect calibration line (Figure 4).

**Table 6:** Expected calibration error(ECE) and mean confidences.

	ANN1		ANN2		ANN3	
	Score	CI	Score	CI	Score	CI
ECE	0.03	(0.02, 0.04)	0.04	(0.03, 0.06)	0.05	(0.04, 0.07)
Outcome Frequency	0.15					
Mean Confidence	0.15		0.15		0.14	
	**XGB1**		**XGB2**		**XGB3**	
	**Score**	**CI**	**Score**	**CI**	**Score**	**CI**
ECE	0.04	(0.03, 0.05)	0.03	(0.03, 0.05)	0.04	(0.03, 0.05)
Outcome Frequency	0.15					
Mean Confidence	0.18		0.18		0.18	
** **	**LR1**		**LR2**		**LR3**	
	**Score**	**CI**	**Score**	**CI**	**Score**	**CI**
ECE	0.02	(0.01, 0.03)	0.02	(0.01, 0.03)	0.02	(0.01, 0.02)
Outcome Frequency	0.15					
Mean Confidence	0.15		0.15		0.15	

**Figure 5:**
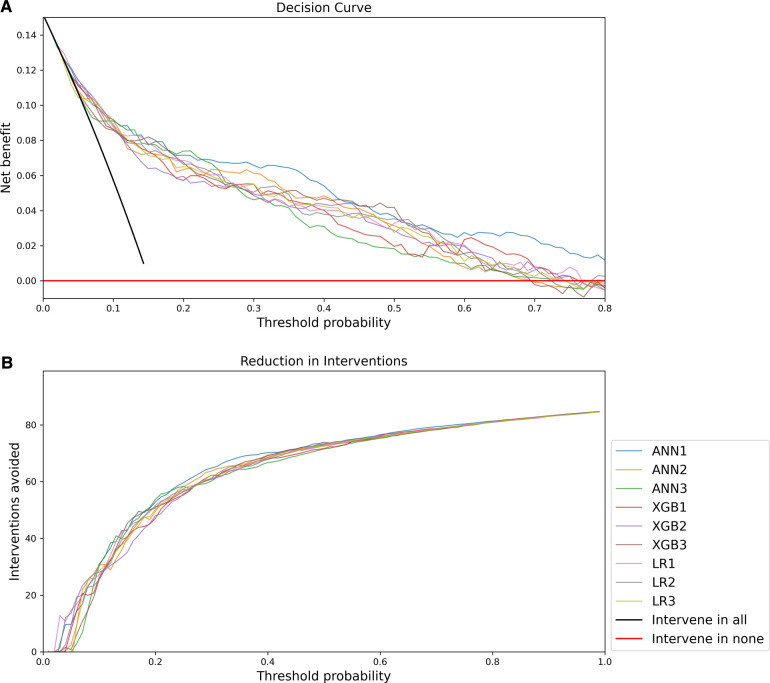
Decision Curve Analysis: (**A**) Net benefit compared to threshold probability; (**B**) Interventions avoided compared to threshold probability.

The decision curve analysis is presented in Figure 5. The net benefit of all models was significantly better than a strategy of intervening in all or no patients, but overall the decision curve analysis favoured ANN1 with a more optimal curve.

## Discussion

This study aimed to develop an artificial neural network model for the prediction of paediatric critical illness (represented by a composite outcome of death before hospital discharge or admission to the PICU) to address avoidable severity of illness and mortality in South African children with a critical illness. In practice, such a model could trigger a range of responses, such as increasing urgency of healthcare worker response, heightened vigilance, further triage and resuscitation, early involvement of senior personnel in case management, or early decision making in the disposition of patients to higher levels of care or centralised hospitals. This engages directly with the challenges identified by Hodkinson et al. in the South African setting (3). The possible integration of such a predictive model within a mobile health (mHealth) platform also offers the potential to create real-time links between the point of care and advanced clinical and digital infrastructure in centralised facilities using simple devices in more resource-constrained settings such as primary healthcare (49).

Methodologically, the development process differed from other applied machine learning studies in this area, such as Aczon et al. (14), Kim et al. (15) and Goto et al. (12), in that these models made use of existing electronic data sets. The lack of such existing data necessitated a dedicated prospective data collection in this study. While the need for a prospective data collection process increased the labour-intensiveness of the study and demand for resources, it also provided an opportunity to integrate clinical domain knowledge within a novel process of documented literature search and Delphi procedure, which allowed tailoring of the data collection process to the specific clinical setting and an explicit description of this process (17). Applications for machine learning research of this nature, invite close interdisciplinary collaboration that allows the integration of machine learning knowledge with clinical domain knowledge, not only of biomedicine but also of clinical context in a fashion that enhances the relevance and applicability of these new methods. The documentation of these processes can be seen to be an important aspect of rigour when publishing reports of such models and demands the investigation of this agenda (50).

All developed models demonstrated satisfactory discrimination. Calibration was variable, with most models demonstrating weak calibration. Given the relative similarity of performance of candidate models, there is likely a significant practical advantage in implementing a parsimonious model in the clinical setting as this would simplify its application, with relatively little loss of clinical benefit.

Decision curve analysis favours the ANN1 over other models. Decision curve analysis also allows incorporation of clinical priorities into the consideration of model performance. In this application, a response such as triggering urgent assessment and resuscitation, involving senior clinical personnel, referral to a higher level of care, or notification and consultation of centralised PICU and emergency medicine services may be directed by such a model. The practical implications and costs of such responses must be weighed against the costs of failing to identify critically ill children timeously. Decision curve analysis in this case suggests that increasing the threshold probability above 0.4 brings about diminished gains in terms of avoided interventions. Given the dire consequences of failure to identify critically ill children and the possible increased costs of healthcare associated with avoidable severity of illness, it may be pragmatic to consider a lower threshold probability for activating such responses.

Given the favourable findings of model performance in cross-validation, an internal validation study is now being undertaken with a view to external validation thereafter. Paediatric research of this nature is novel in the South African setting, with one similar publication from this centre to date (16). This study furthers the research agenda for machine learning research and applied machine learning in South African healthcare.

## Conclusion

All 9 models demonstrated satisfactory discrimination and weak calibration in cross-validation. Overall assessment, including decision curve analysis, however, favours the ANN1 over other models. Decision curve analysis provides useful insights into how such a model may be implemented at different probability thresholds considering clinical preference and judgement. While ANN1 demonstrated the best performance in this data, other data sets using fewer features also provided adequate performance. This has important practical implications for implementation in clinical settings.

There is still a significant need to investigate machine learning research and clinical application in South Africa and other similar settings. In this study, a documented domain knowledge elicitation process has been combined with machine learning methods to develop models directed at identifying critically ill children. Model performance will be further evaluated in internal and external validation studies.

## Data Availability

The original contributions presented in the study are included in the article/**Supplementary Material**, further inquiries can be directed to the corresponding author/s.
