# Supplementary material for: Development of artificial neural network models for paediatric critical illness in South Africa
Source: Front Pediatr. 2022 Nov 15;10:1008840. doi: 10.3389/fped.2022.1008840 (PMC9705750; doi:10.3389/fped.2022.1008840)
Supplement: Supplementary file 1 [file Datasheet1.docx]

**Supplementary Table 1: Initial Feature Set**

| Continuous Features | |
| --- | --- |
|  | **Unit** |
| Age | Months |
| Respiratory Rate | Frequency |
| SPO_2_ | % |
| Pulse | Frequency |
| Systolic Blood Pressure | mmHg |
| Diastolic Blood Pressure | mmHg |
| Capillary Refill Time | Seconds |
| Weight | kg |
| Height | cm |
| Temperature | °C |
| Glucose | mmol/L |
| Categorical Features | |
|  | **Categories** |
| Deep Breathing | No  Yes |
| Weak Pulse | No  Yes |
| Level of Consciousness | Alert  Prostrate  Coma |
| AVPU Scale | Alert  Verbal  Pain  Unresponsive |
| Unable to Feed | No  Yes |
| Respiratory Distress | No  Yes |
| Jaundice | No  Yes |
| Seizures | No  Yes |
| Respiratory Support | Room Air  Nasal Cannula  Intubated |
| HIV Infection | Unexposed  Exposed, uninfected  Infected  *Untreated*  *Treatment <3months*  *Treatment >= 3 months*  *Treatment Interrupted*  Unknown |
| Outcome | Death  PICU admission  Combined outcome |

**Supplementary Table 2: Cross Validation ROC AUC scores with 95% Confidence Intervals**

|  | ANN1 | CI | XGB1 | CI | LR1 | CI |
| --- | --- | --- | --- | --- | --- | --- |
| Fold 1 | 0.83 | 0.73 - 0.93 | 0.82 | 0.68 - 0.93 | 0.83 | 0.70 - 0.92 |
| Fold 2 | 0.92 | 0.84 - 0.97 | 0.86 | 0.76 - 0.94 | 0.87 | 0.78 - 0.95 |
| Fold 3 | 0.85 | 0.74 - 0.93 | 0.81 | 0.70 - 0.91 | 0.81 | 0.71 - 0.90 |
| Fold 4 | 0.75 | 0.62 - 0.87 | 0.73 | 0.60 - 0.84 | 0.79 | 0.69 - 0.89 |
| Fold 5 | 0.87 | 0.79 - 0.94 | 0.84 | 0.75 - 0.92 | 0.85 | 0.75 - 0.93 |
|  | **ANN2** | **CI** | **XGB2** | **CI** | **LR2** | **CI** |
| Fold 1 | 0.80 | 0.68 - 0.90 | 0.84 | 0.75 - 0.93 | 0.85 | 0.72 - 0.92 |
| Fold 2 | 0.89 | 0.79 - 0.96 | 0.85 | 0.75 - 0.94 | 0.87 | 0.77 - 0.95 |
| Fold 3 | 0.84 | 0.73 - 0.93 | 0.82 | 0.72 - 0.91 | 0.82 | 0.72 - 0.90 |
| Fold 4 | 0.73 | 0.61 - 0.85 | 0.74 | 0.609 - 0.851 | 0.77 | 0.66 - 0.87 |
| Fold 5 | 0.87 | 0.79 - 0.94 | 0.83 | 0.731 - 0.91 | 0.85 | 0.74 - 0.94 |
|  | **ANN3** | **CI** | **XGB3** | **CI** | **LR3** | **CI** |
| Fold 1 | 0.82 | 0.70 - 0.92 | 0.79 | 0.68 - 0.89 | 0.83 | 0.73 - 0.92 |
| Fold 2 | 0.88 | 0.77 - 0.95 | 0.87 | 0.75 - 0.96 | 0.84 | 0.711 - 0.94 |
| Fold 3 | 0.79 | 0.65 - 0.91 | 0.81 | 0.68 - 0.91 | 0.76 | 0.62 - 0.89 |
| Fold 4 | 0.80 | 0.69 - 0.89 | 0.77 | 0.65 - 0.89 | 0.75 | 0.62 - 0.87 |
| Fold 5 | 0.84 | 0.72 - 0.93 | 0.80 | 0.69 - 0.91 | 0.83 | 0.70 - 0.93 |

**Supplementary Table 3: Cross Validation PRC AUC scores with 95% Confidence Intervals**

|  | ANN1 | CI | XGB1 | CI | LR1 | CI |
| --- | --- | --- | --- | --- | --- | --- |
| Fold 1 | 0.49 | 0.42 - 0.91 | 0.58 | 0.39 - 0.77 | 0.53 | 0.36 - 0.75 |
| Fold 2 | 0.72 | 0.62 - 0.97 | 0.57 | 0.39 - 0.80 | 0.67 | 0.46 - 0.83 |
| Fold 3 | 0.64 | 0.54 - 0.92 | 0.54 | 0.34 - 0.71 | 0.49 | 0.31 - 0.68 |
| Fold 4 | 0.42 | 0.28 - 0.85 | 0.40 | 0.23 - 0.63 | 0.44 | 0.27 - 0.67 |
| Fold 5 | 0.66 | 0.55 - 0.94 | 0.62 | 0.41 - 0.80 | 0.62 | 0.42 - 0.80 |
|  | **ANN2** | **CI** | **XGB2** | **CI** | **LR2** | **CI** |
| Fold 1 | 0.49 | 0.36 - 0.89 | 0.59 | 0.41 - 0.77 | 0.56 | 0.37 - 0.75 |
| Fold 2 | 0.72 | 0.56 - 0.95 | 0.59 | 0.38 - 0.80 | 0.64 | 0.43 - 0.81 |
| Fold 3 | 0.64 | 0.49 - 0.92 | 0.54 | 0.34 - 0.71 | 0.52 | 0.33 - 0.71 |
| Fold 4 | 0.42 | 0.27 - 0.83 | 0.43 | 0.24 - 0.65 | 0.42 | 0.25 - 0.64 |
| Fold 5 | 0.66 | 0.50 - 0.93 | 0.61 | 0.40 - 0.79 | 0.65 | 0.45 - 0.82 |
|  | **ANN3** | **CI** | **XGB3** | **CI** | **LR3** | **CI** |
| Fold 1 | 0.51 | 0.36 - 0.90 | 0.54 | 0.36 - 0.71 | 0.53 | 0.34 - 0.72 |
| Fold 2 | 0.68 | 0.52 - 0.95 | 0.64 | 0.44 - 0.83 | 0.64 | 0.43 - 0.82 |
| Fold 3 | 0.58 | 0.41 - 0.89 | 0.55 | 0.36 - 0.78 | 0.51 | 0.31 - 0.72 |
| Fold 4 | 0.42 | 0.29 - 0.88 | 0.47 | 0.27 - 0.68 | 0.42 | 0.26 - 0.64 |
| Fold 5 | 0.62 | 0.43 - 0.92 | 0.57 | 0.37 - 0.78 | 0.62 | 0.42 - 0.81 |
