# Supplementary figures and images for: Development of artificial neural network models for paediatric critical illness in South Africa
Source: Front Pediatr. 2022 Nov 15;10:1008840. doi: 10.3389/fped.2022.1008840 (PMC9705750; doi:10.3389/fped.2022.1008840)

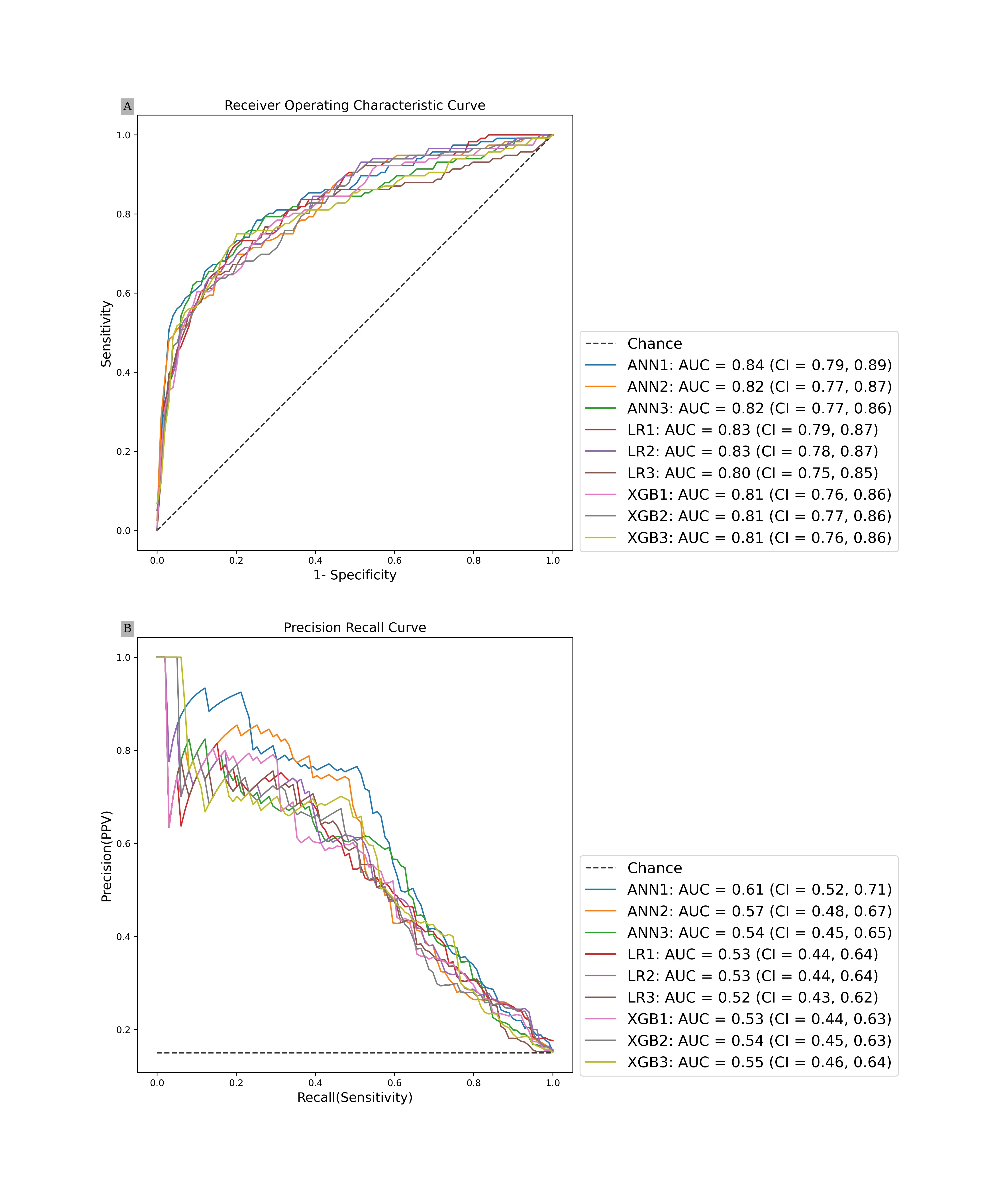

Supplement: Supplementary file 2 [file Image1.jpeg]
